# Systematic Review and Meta-analysis of the Prevalence of Group A Streptococcal *emm* Clusters in Africa To Inform Vaccine Development

**DOI:** 10.1128/mSphere.00429-20

**Published:** 2020-07-15

**Authors:** Taariq Salie, Kelin Engel, Annesinah Moloi, Babu Muhamed, James B. Dale, Mark E. Engel

**Affiliations:** a AFROStrep Research Group, Department of Medicine, Faculty of Health Sciences, University of Cape Town & Groote Schuur Hospital, Cape Town, South Africa; b Health Systems Research Unit, South African Medical Research Council, Cape Town, South Africa; c Children's National Health System, Division of Cardiology, Washington, DC, USA; d Division of Infectious Diseases, Department of Medicine, University of Tennessee Health Science Center, Memphis, Tennessee, USA; University of Missouri—Kansas City School of Medicine

**Keywords:** group A *Streptococcus*, *Streptococcus pyogenes*, GAS, M protein, *emm* clustering system, epidemiology, surveillance, vaccine, systematic review, Strep A

## Abstract

An *emm*-cluster based system was proposed as a standard typing scheme to facilitate and enhance future studies of group A *Streptococcus* (GAS) epidemiological surveillance, M protein function, and vaccine development strategies. We provide an evidence-based distribution of GAS *emm* clusters in Africa and assess the potential coverage of the new 30-valent vaccine in terms of an *emm* cluster-based approach. Two reviewers independently assessed studies retrieved from a comprehensive search and extracted relevant data. Meta-analyses were performed (random-effects model) to aggregate *emm* cluster prevalence estimates. Eight studies (*n* = 1,595 isolates) revealed the predominant *emm* clusters as E6 (18%; 95% confidence interval [CI], 12.6% to 24.0%), followed by E3 (14%; 95% CI, 11.2% to 17.4%) and E4 (13%; 95% CI, 9.5% to 16.0%). There was negligible variation in *emm* clusters with regard to regions, age, and socioeconomic status across the continent. Considering an *emm* cluster-based vaccine strategy, which assumes cross-protection within clusters, the 30-valent vaccine currently in clinical development would provide hypothetical coverage to 80.3% of isolates in Africa. This systematic review indicates the most predominant GAS *emm* cluster in Africa is E6 followed by E3, E4, and D4. The current 30-valent vaccine would provide considerable coverage across the diversity of *emm* cluster types in Africa. Future efforts could be directed toward estimating the overall potential coverage of the new 30-valent vaccine based on cross-opsonization studies with representative panels of GAS isolates from populations at highest risk for GAS diseases.

**IMPORTANCE** Low vaccine coverage is of grave public health concern, particularly in developing countries where epidemiological data are often absent. To inform vaccine development for group A *Streptococcus* (GAS), we report on the epidemiology of the M protein *emm* clusters from GAS infections in Africa, where GAS-related illnesses and their sequelae, including rheumatic fever and rheumatic heart disease, are of a high burden. This first report of *emm* clusters across the continent indicates a high probably of coverage by the M protein-based vaccine currently undergoing testing were an *emm*-cluster based approach to be used.

## Abstract (precis)

Low vaccine coverage is of grave public health concern, particularly in developing countries where epidemiological data are often absent. To inform vaccine development for group A *Streptococcus* (GAS), we report on the epidemiology of the M protein *emm* clusters from GAS infections in Africa, where GAS-related illnesses and their sequelae, including rheumatic fever and rheumatic heart disease, are of a high burden. This first report of *emm* clusters across the continent indicates a high probably of coverage by the M protein-based vaccine currently undergoing testing were an *emm*-cluster based approach to be used.

## INTRODUCTION

Group A *Streptococcus* (GAS) causes a range of human infections, including pharyngitis and impetigo, which can lead to nonsuppurative (immune-mediated) sequelae such as acute rheumatic fever (ARF) and rheumatic heart disease (RHD) if not properly managed (1). Additionally, GAS has the ability to cause invasive infection, such as sepsis, necrotizing fasciitis, pneumonia, and streptococcal toxic shock syndrome (STSS) in children and adults (2), with a high fatality rate; furthermore, it is a leading cause of maternal death in some regions (3). Noninvasive GAS infections mostly affect young children and women living in developing countries (4), while severe invasive infections are both more common in adults (and increases with age) and more common in men than in women (5). The estimated symptomatic GAS pharyngitis annual incidence rate is 0.4 cases per person-year, with more than 423 million cases in children residing in developing countries (1).

The dire complications and huge economic burden of GAS infections and their sequelae, with an estimated global annual incidence of 616 million pharyngitis and 1.78 million severe cases (1), support the urgent need for an effective vaccine that would provide broad coverage of circulating GAS strains (6). One of the GAS vaccine strategies targets the M protein on the bacterial surface, which has thermal stability, antiphagocytic properties, and the capacity to evoke antibodies with the greatest bactericidal activity (7). The hypervariable N-terminal region of the M protein displays extensive nucleotide differences, thus giving rise to various M protein amino acid sequences which imparts serological specificity (8). The 5′ *emm* sequence encoding the mature protein is the basis for categorizing different GAS strains through molecular typing methods, which aid in defining the epidemiology of GAS infections.

A 30-valent N-terminal M protein-based vaccine (9) is undergoing clinical trials (10). The vaccine composition was based on extensive GAS surveillance data from developed regions such as the United States and Europe on those isolates that are involved in invasive disease, those associated with superficial infections, and those causing autoimmune diseases (9, 11). However, given the >200 GAS *emm* types characterized to date (12), the absence of highly prevalent GAS subtypes in the current vaccine formulation may diminish the coverage of at-risk populations outside western countries.

An *emm* clustering system was introduced by Sanderson-Smith and colleagues that phylogenetically analyzed the whole-M protein sequences, organizing *emm* types into clusters that have the same or similar sequences and host protein binding properties (13). This proposed classification allows for the previously identified GAS *emm* types to be categorized into 48 discrete *emm* clusters (13), where more than one *emm* type may be contained within a cluster (Table 1). The *emm* cluster system complements the *emm* typing system, which may serve to enhance studies relating to M protein function, streptococcal virulence, epidemiological surveillance, and vaccine development (13). *emm* clusters E1 to E6 were placed into clade X, binding to immunoglobulin and C4BP. While A-C1 through A-C5 and D1 to D5 were grouped into clade Y, with a host protein tropism toward plasminogen and fibrinogen.

**TABLE 1:** *emm* clusters and their corresponding *emm* types^*a*^

*emm* types	*emm* cluster
4, 60, 78, 165 (st11014), 176 (st213)	E1
13, 27, 50 (50/62), 66, 68, 76, 90, 92, 96, 104, 106, 110, 117, 166 (st1207), 168 (st1389)	E2
9, 15, 25, 44 (44/61), 49, 58, 79, 82, 87, 103, 107, 113, 118, 144 (stknb1), 180 (st2460), 183 (st2904), 209 (st6735), 219 (st9505), 231 (stNS292)	E3
2, 8, 22, 28, 73, 77, 84, 88, 89, 102, 109, 112, 114, 124, 169 (st1731), 175 (st212), 232 (stNS554)	E4
34, 51, 134 (st2105), 137 (st465), 170 (st1815), 174 (st211), 205 (st5282)	E5
11, 42, 48, 59, 63, 65 (65/69), 67, 75, 81, 85, 94, 99, 139 (st7323), 158 (stxh1), 172 (st2037), 177 (st2147), 182 (st2861UK), 191 (st369)	E6
164 (st106), 185 (st2917), 211 (st7406), 236 (sts104)	Single protein *emm*-cluster clade X
36, 54, 207 (st6030)	D1
32, 71, 100, 115, 213 (st7700)	D2
123, 217 (st809)	D3
33, 41, 43, 52, 53, 56, 56.2 (st3850), 64, 70, 72, 80, 83, 86, 91, 93, 98, 101, 108, 116, 119, 120, 121, 178 (st22), 186 (st2940), 192 (st3757), 194 (st38), 208 (st62), 223 (stD432), 224 (stD631), 225 (stD633), 230 (stNS1033), 242 (st2926)	D4
97, 157 (stn165), 184 (st2911)	D5
46, 142 (st818)	A-C1
30, 197 (st4119)	A-C2
1, 163 (st412), 227 (stil103), 238, 239	A-C3
12, 39, 193 (st3765), 228 (stil62), 229 (stmd216)	A-C4
3, 31, 133 (st1692)	A-C5
5, 6, 14, 17, 18, 19, 23, 24, 26, 29, 37, 38 (38/40), 47, 57, 74, 105, 122, 140 (st7395), 179 (st221), 218 (st854), 233 (stNS90), 234 (stpa57)	Single protein *emm*-cluster clade Y
55, 95, 111, 215 (st804), 221 (stCK249), 222 (stCK401)	Single protein *emm*-cluster outlier

a Reprinted from Sanderson-Smith et al. (13) with permission from the publisher.

To date, significant *emm* cluster data have been produced through *emm* typing of GAS, with recent studies reporting on *emm* cluster epidemiology. Shulman et al. documented the most prevalent *emm* clusters in the United States as E4 (27.16%), A-C3 (17.78%), and A-C4 (17.56%) among 7,040 isolates (14). The prevalence of *emm* clusters in three Pacific countries, *viz.* Australia, Fiji, and New Caledonia, illustrated that 70% to 84% of clusters from isolates were shared, as opposed to comparison of *emm* types having only 14% to 30% commonality between countries (15). In a third study by Chiang-Ni et al. in Taiwan, an analysis of both invasive and noninvasive strains revealed that cluster E6 was associated with both types of infections, while clusters D4, E2, and E3 were responsible for causing invasive isolates in their population (16). Recently, Frost et al. demonstrated that M type-specific and cross-reactive immune responses frequently align with *emm* clusters, raising new opportunities to design multivalent vaccines with broad coverage (17).

A thorough review of *emm* cluster data from Africa has not yet been undertaken. A study that aggregates the African data on clusters is essential to contribute to the growing literature in efforts to develop a GAS vaccine on a global scale, particularly in low-income countries where the burden of disease is greatest. Therefore, this review sought to provide an evidence-based distribution of GAS *emm* clusters in Africa.

## RESULTS

**FIG 1:**
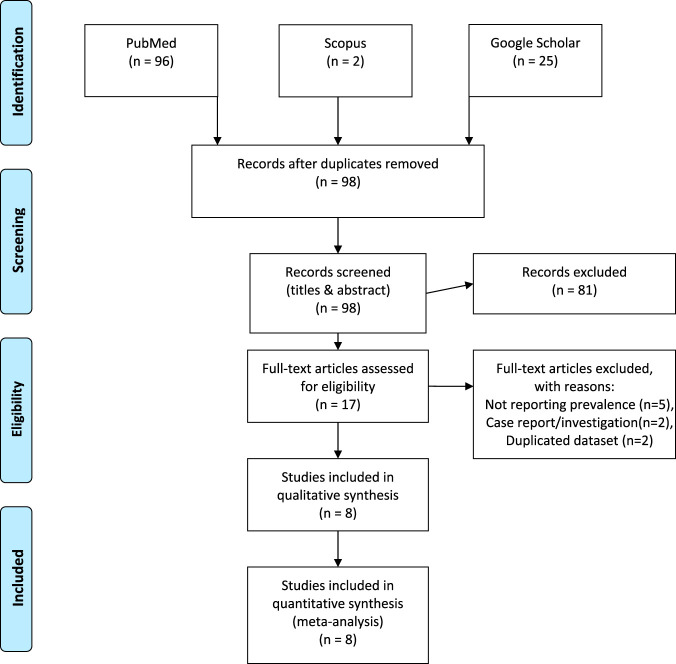
Schematic PRISMA flow diagram of the literature search. Figure is modeled after reference 18.

The literature search for articles was reported according to the Preferred Reporting Items for Systematic reviews and Meta-Analysis (PRISMA) statement (18). Figure 1 details the search results with the retrieval of 121 articles for consideration from the respective electronic databases. After title screening and the removal of duplicates, we excluded 23 articles. We reviewed the remaining abstracts and excluded a further 81 articles, leaving 17 articles requiring full-text evaluation. Finally, eight articles met the inclusion criteria and were included in the review. A list of the excluded studies with reasons are detailed in Table S4 in reference 19.

### Characteristics of included studies.

The included articles were published between 2004 and 2019 with sample sizes ranging from 43 to 396 total isolates. Of these, two articles had cross-sectional study designs, while the remaining studies took a prospective passive surveillance approach. The ages of participants included in the studies were also recorded; six articles studied isolates obtained from children (range, 0 to 18 years old) and two studied patients of all ages. Studies were conducted in local and university hospitals, clinics, outpatient departments, and schools situated in the study areas (Table 2).

**TABLE 2:** Characteristics of included studies

Study ID^*a*^	Country	Design	Setting (local, social context)	Socioeconomic setting	Population description	Inclusion criteria^*b*^	Age (yrs)
Abdissa 2005	Ethiopia	Cross-sectional	Public primary schools situated in Addis Ababa, Gondar, and Dire Dawa	Low income	Healthy children attending public primary schools	Healthy school children in area	4–16
Barth 2019	South Africa	Prospective passive surveillance	Groote Schuur Hospital in Cape Town	Upper middle income	Children attending public clinics	Patients with confirmed GAS infection (INV and NINV)	All
Engel 2014	South Africa	Cross-sectional	Langa Clinic, Vanguard Community Health Centre	Upper middle income	Children attending public clinics	Children with sore throats	3–15
Hraoui 2011	Tunisia	Passive surveillance	Microbiology lab of Charles Nicolle University Hospital in Tunis	Lower middle income	Patients attending the local hospital	Patients with confirmed GAS infection (INV and NINV)	All
Mzoughi 2004	Tunisia	Prospective surveillance	Farhat Hached Hospital & Centre PMI, Sousse	Lower middle income	Pediatric outpatients attending clinic	Children with pharyngitis	2–8
Seale 2016	Kenya	Prospective surveillance	Kenya Medical Research Institute, Kilifi County Hospital	Lower middle income	Children admitted for medical care at the hospital	Children located in the area with confirmed INV	0–12
Tapia 2016	Mali	Prospective surveillance	Four public schools in Djikoroni Para and Sebenikoro	Low income	Children attending 1 of the 4 schools	Children with sore throats	5–16
Tewodros 2005	Ethiopia	Prospective surveillance	Black Lion Hospital and 3 elementary schools in Addis Ababa	Low income	Pediatric patients attending the hospital and schools within area	Healthy children at schools & those with confirmed ARF, RHD, APSGN, and impetigo	<18

a ID, identifier (first author surname and year of publication).

b INV, invasive GAS; NINV, noninvasive GAS; ARF, acute rheumatic fever; APSGN, acute poststreptococcal glomerulonephritis.

The country of each article was recorded, with 2 articles obtained from Ethiopia (20, 21), two from South Africa (22, 23), two from Tunisia (24, 25), and one article each from Kenya (26) and Mali (27). All the studies included in this review made use of the gold-standard *emm*-typing molecular procedure proposed by Beall et al. (28) and the CDC (29).

### Prevalence of GAS *emm* clusters.

**FIG 2:**
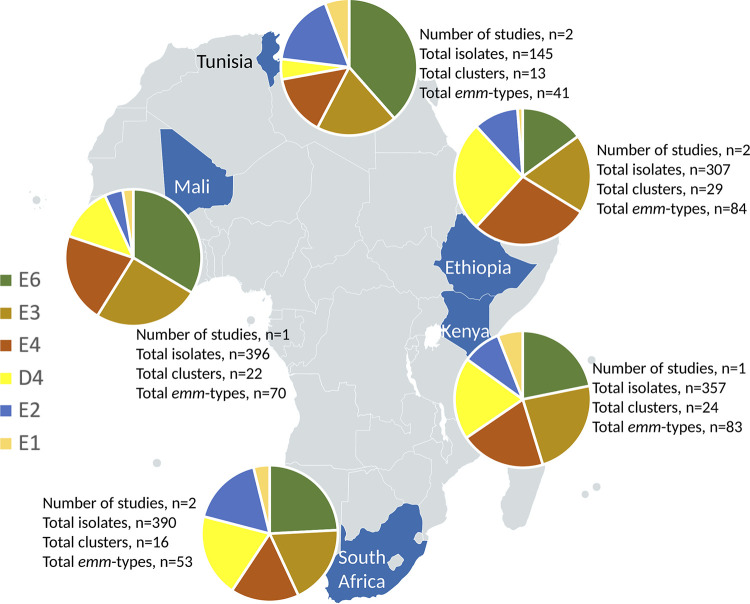
The five countries included in the review, representing the most abundant *emm* clusters.

Five countries within Africa contributed *emm* cluster data to this review (Fig. 2). The final data set included 1,532 isolates representing 126 heterologous *emm* types. Of these, 1,291 isolates, comprising 96 *emm* types, constituted 16 *emm* clusters. Of those remaining, 186 isolates contained 18 single-isolate *emm* clusters (15 *emm* types [143 isolates] representing 15 *emm* clusters belonging to clade Y, while *emm*55, *emm*95, and *emm*111 constituted outliers [43 isolates]) (Table 3). The remaining 12 *emm* types (55 isolates) are among those as yet not classified. The predominant clusters were E6 with 294 isolates (18.4%), followed by E3 (*n* = 243, 15.2%) and E4 (*n* = 225, 14.1%). The *emm* clusters with the least number of isolates are D1 and E5, each having a single isolate. *emm* cluster A-C1 was not represented. Sixty-three isolates were reported as “untypeable” by authors, thus not assigned an *emm* type, or as an “old” *emm* type that does not correspond with the CDC classification.

**TABLE 3:** *emm* cluster distribution, representing the five countries included in the review and their respective isolate counts

*emm* cluster	No. of isolates					Total no. (%)
	Ethiopia	Kenya	Mali	South Africa	Tunisia	
Clade Y						
A-C1	0	0	0	0	0	0 (0.0)
A-C2	4	1	0	0	0	5 (0.3)
A-C3	12	3	1	11	9	36 (2.3)
A-C4	17	3	0	13	7	40 (2.6)
A-C5	12	0	1	2	4	19 (1.2)
D1	1	0	0	0	0	1 (0.1)
D2	10	4	6	1	0	21 (1.4)
D3	1	3	7	0	0	11 (0.7)
D4	42	49	36	67	5	199 (13.0)
D5	3	3	6	9	0	21 (1.4)
Clade X						
E1	2	15	7	13	6	47 (3.1)
E2	17	23	12	58	18	128 (8.4)
E3	30	59	70	64	20	243 (15.9)
E4	45	51	59	55	15	225 (14.7)
E5	1	0	0	0	0	1 (0.1)
E6	24	55	93	82	40	294 (19.2)
Single-type clusters						
Clade Y						
M5	7	0	0	0	0	7 (0.5)
M6	4	0	0	5	4	13 (0.8)
M14	1	0	0	0	1	2 (0.1)
M18	8	12	17	0	4	41 (2.7)
M19	1	2	7	1	0	11 (0.7)
M26	0	1	0	0	1	2 (0.1)
M29	4	0	0	0	0	4 (0.3)
M38	2	1	0	0	0	3 (0.2)
M57	1	1	0	0	0	2 (0.1)
M74	12	4	8	1	0	25 (1.6)
M105	2	0	1	0	0	3 (0.2)
M122	0	2	8	0	0	10 (0.7)
M179	1	10	1	0	0	12 (0.8)
M218	2	3	2	0	0	7 (0.5)
M234	0	0	0	1	0	1 (0.1)
Outliers						
M55	1	6	16	0	0	23 (1.5)
M95	2	2	7	2	0	13 (0.8)
M111	0	4	3	0	0	7 (0.5)
No *emm* cluster^*a*^	20	11	21	3	0	55 (3.6)
Total^*b*^	307	357	396	390	145	1532

a Those *emm* types that has not been assigned to a particular clade by Sanderson-Smith et al. (13).

b Sixty-three isolates were “untypeable” by the author and was not assigned an *emm* type, or an “old” *emm* type that does not correspond with the CDC classification.

There were four regions represented across Africa. Variation of clusters across the regions was not remarkable. Interestingly, single-isolate cluster M55 was specific to Mali in West Africa, containing 16 isolates. The highest single-isolate cluster, M18 (*n* = 41 isolates), was not represented in South Africa. Where age of participants in studies was provided, there was no difference among children (<18 years of age) and adults in terms of cluster prevalence. By clinical manifestation, isolates from invasive disease numbered 516 (32.4%) (Figure S1 in reference 19), with the most prevalent clusters being E3 (*n* = 91 isolates) followed by E6 (*n* = 82) and D4 (*n* = 77). No variation in *emm* clusters by socioeconomic status was apparent.

### Overall prevalence of GAS *emm* clusters represented by the *emm* types included in the 30-valent vaccine.

**FIG 3:**
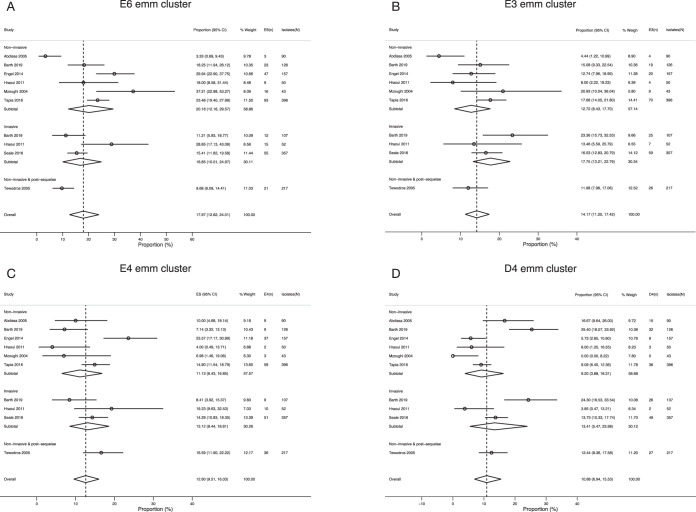
Forest plots showing the combined prevalence estimates of the four most abundant *emm* clusters of all included studies. (A) E3 *emm* cluster; (B) E6 *emm* cluster; (C) E4 *emm* cluster; (D) D4 *emm* cluster. Studies are represented by author, year of publication, effect size (ES) (proportion [95% CI]). *n*, number of isolates in cluster; *N*, total number of isolates in study.

Cluster E6 was the most represented *emm* cluster (17.97%; 95% confidence interval [CI], 12.6% to 24.0%) among African isolates (Fig. 3A). This was followed by E3 (14.17%; 95% CI, 11.2% to 17.4%), E4 (12.6% of isolates; 95% CI, 9.5% to 16.0%), D4 (10.88%; 95% CI, 6.9% to 15.5%) (Fig. 3B to D), and E2 (9.12%; 95% CI, 4.6% to 14.9%) of isolates. Clusters A-C3, A-C4, A-C5, and E1 each had an effect size of ∼2%. Isolates from invasive disease were abundant in clusters D4, E2, E3, and E4, while only E6 had a preponderance of strains from noninvasive disease (Table 4).

**TABLE 4:** Summary of the meta-analyses completed^*a*^

Cluster	*emm* type(s) in vaccine	*emm* types in African isolates^*b*^	Combined prevalence (95% CI)	No. (%) of *emm* type isolates			
				NINV	INV	N/D	Total
E6	11, 75, 81	Prot: 11, **48**, **59**, **65**, 75, 81, **85**, **94** (*n* = 235 [79,9%]); N/P: 42, 63, 67, 99, 158, 182; NINV: *n* = 160/191 (83.8%); INV: *n* = 59/82 (72.0%)	18.0 (12.62–24.01)	191 (65.0)	82 (27.9)	21 (7.1)	294
E3	44, 49, 58, 87,118	Prot: **9**, **15**, 44, 49, 58, **79**, 87, 118 (*n* = 130 [53.5%]); N/P: 25, 61, 82, 103, 113, 180, 183, 209; NINV: *n* = 73/126 (57.9%); INV: *n* = 47/91 (51.6%)	14.2 (11.20–17.42)	126 (51.9)	91 (37.4)	26 (10.7)	243
E4	2, 22, 28, 73, 77, 89, 114	Prot: 2, **8**, 22, 28, 73, 77, 89, **109**, 114 (*n* = 202 [89.8%]); N/P: 84, 112, 124, 175, 225; NINV: *n* = 115/119 (96.6%); INV: *n* = 52/70 (74.3%)	12.6 (9.51–16.03)	119 (52.9)	70 (31.1)	36 (16.0)	225
D4	83	Prot: **33**, 83 (*n* = 15 [7.5%]); N/P: 41, 43, 53, 56, 64, 70, 80, 86, 93, 98, 116, 119, 121, 186, 192, 208, 223, 224, 230; NINV: *n* = 5/95 (5.3%); INV: *n* = 9/77 (11.7%)	10.9 (6.94–15.53)	95 (47.7)	77 (38.7)	27 (13.6)	199
E2	92	Prot: **66**, **68**, **76**, 92, **102** (*n* = 92 [71.9%]); N/P: 50, 90, 104, 106, 110, 168; NINV: *n* = 49/63 (77.8%); INV: *n* = 38/55 (69.1%)	9.1 (4.61–14.86)	63 (49.2)	55 (43.0)	10 (7.8)	128
E1	4, 78	Prot: 4, 78 (*n* = 31 [66.0%]); N/P: 60, 165; NINV: *n* = 22/28 (78.6%); INV: *n* = 8/17 (47.1%)	1.9 (0.62–3.81)	28 (59.6)	17 (36.2)	2 (4.3)	47
A-C4	12	Prot: 12 (*n* =35 [87.5%]); I (*n* = 3 [42.9%]); N/P: 39, 193, 229; NINV: *n* = 17/18 (94.4%); INV: *n* = 3/7 (42.9%)	2.1 (0.37–4.81)	18 (45.0)	7 (17.5)	15 (38.0)	40
A-C3	1	Prot: 1 (*n* = 32 [88.9%]); N/P: 238; NINV: *n* = 21/22 (95.5%); INV: *n* = 1/4 (25.0%)	2.0 (0.46–4.31)	22 (61.1)	4 (11.1)	10 (27.8)	36
A-C5	3	Prot: 3 (*n* = 19 [100%]); N/P: NA; NINV: *n* = 17/17 (100%); INV: *n* = 2/2 (100%)	0.9 (0.01–2.72)	17 (89.5)	2 (10.5)	0 (0.0)	19

a Three hundred one isolates (19.6%) comprising 39 *emm* types are not included in any of the *emm* clusters contained in the 30-valent vaccine; these include 60 isolates representing seven *emm* clusters, 186 isolates representing single-isolate clusters, and 55 isolates that were not classified as according to Sanderson-Smith et al. (13).

b Bold *emm* types represent cross-opsonized nonvaccine types. The study completed by Tewodros and Kronvall (21) did not clearly differentiate its *emm* types according to clinical manifestation. Combined prevalence calculated with Mantel-Haenszel (M-H) meta-analysis procedures. NINV, noninvasive; INV, invasive; Prot, protected; N/P, not protected; N/D, not differentiated; NA, none.

### M protein vaccine coverage.

**FIG 4:**
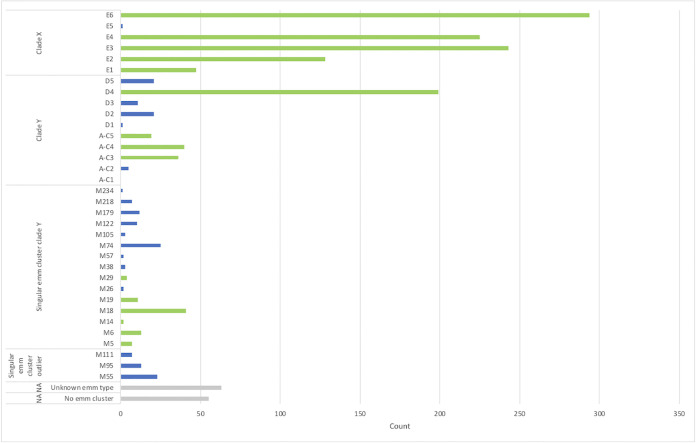
Count of isolates within *emm* clusters from African studies included in this systematic review. Green bars indicate *emm* clusters represented in the 30-valent vaccine. Blue bars represent *emm* clusters not included in the vaccine. Gray bars represent isolates unassigned to a cluster or were “untypeable” according to the authors’ report. Numbers represent the count of isolates across all studies.

Slightly more than 80 percent (80.3%) of African GAS isolates are classified in clusters included in the 30-valent vaccine (Fig. 4). However, based on *emm* types within the vaccine, together with *emm* types known to be cross-opsonized, the number of African GAS isolates that potentially could be covered by the 30-valent vaccine amounts to 892 of the 1,532 isolates, corresponding to 58.22% (comprising 599 vaccine-type *emm* types and 293 nonvaccine *emm* types) (9). For the *emm* types representing the remaining 640 isolates (41.78%), there is either no information yet available about possible cross-protection or the *emm* types would not be expected to cross-react with the 30-valent vaccine antisera because they are in single-*emm* clusters or in clusters not represented by the vaccine. Interestingly, isolates classified as *emm*30 (AC-2), *emm*36 (D1), *emm*51 (E5), and *emm*97 (D5), despite not being in a cluster represented in the vaccine, are nevertheless afforded cross-protection.

With regard to invasive *emm* types in Africa, the overall potential coverage of the vaccine (based on published results of cross-opsonization) was 54.1% for clusters included in the meta-analyses (Table 4). More specifically, coverage for clusters E6, E4, and E2 ranged from 69% to 74% of invasive isolates; only ∼50% of strains would be protected in E3, and coverage for the remaining clusters was below 47% except for A-C5 (100% coverage), as there were only two invasive strains reported. Interestingly, the 30-valent vaccine would potentially only provide 12% coverage to invasive isolates belonging to the fourth highest cluster, D4 (*n* = 28 *emm* types).

### Assessment of risk of bias of included studies.

The results from the assessment is portrayed in the Table 5, with two studies having a low risk of bias (23, 27) and the remaining studies being of moderate bias. All the articles narrowed down their target population by focusing on a specific age group, clinical manifestation, or geographical area. The data in all included studies were collected directly from the study participants as opposed to by proxy, confirming the reliability of sample collection and patient demographics. The included studies clearly described the phenotypes of patients, providing an acceptable case definition or diagnostic algorithm. Studies focusing on invasive GAS infections isolated GAS from normally sterile sites such as blood, cerebrospinal fluid, joints, bones, or synovium among others. Noninvasive GAS was isolated from skin or throat via swabs of the infected area.

**TABLE 5:** Summary of risk of bias assessment

Study ID	Risk of bias:^*a*^							Quality score	Risk of bias
	1	2	3	4	5	6	7		
Abdissa 2005	0	1	1	1	1	1	0	5	Moderate
Barth 2019	0	1	1	1	1	1	1	6	Low
Engel 2014	0	1	1	1	1	1	0	5	Moderate
Hraoui 2011	0	1	1	1	1	1	0	5	Moderate
Mzoughi 2004	0	1	1	1	NCS^*b*^	1	0	4	Moderate
Seale 2016	0	1	1	1	1	1	0	5	Moderate
Tapia 2016	0	1	1	1	1	1	1	6	Low
Tewodros 2005	0	1	1	1	1	1	0	5	Moderate

a Risk of bias categories: 1 and 2, representativeness of population; 3 and 6, data collection; 4, case definitions; 5, study instrument reliability; 7, limitations.

b NCS, not clearly stated.

## DISCUSSION

This systematic review provides evidence for the distribution of *emm* clusters of GAS in Africa, specifically focusing on the epidemiological differences within Africa and added value of the *emm* clustering system in assisting with vaccine development. Using prevalence data obtained from eight studies representing five countries within Africa, this report identified the predominant *emm* clusters in Africa, namely, E6 followed by E3, E4, and D4. We further report that the *emm* clusters contained in the current 30-valent vaccine could provide slightly more than 80% coverage across the diversity of *emm* cluster types in Africa.

Comparing results to other *emm* clustering epidemiology studies, it is clear that the dominant *emm* clusters vary between regions. Only cluster E3 in the present study is common with the Pacific region (15). When comparing the current data to that from the United States, E4, the highest cluster in the United States, is the third highest cluster, whereas A-C3 and A-C4 together only amount to ∼2% of the total strains isolated in Africa (14). This study emphasizes that *emm* clusters E6, E3, and D4, prevalent in the African populations where the burden of GAS infections is highest (30), should take prominence alongside clusters E4, A-C3, and A-C4.

We note that there are a number of *emm* clusters containing a single *emm* type, as they do not share similar binding properties or sequences. Also, there are many *emm* types that have not as yet been categorized into a particular cluster, as this may be due to their recent emergence after the proposed cluster system. This should be the focus of future studies in which more associations with human host protein binding could be tested to determine any other similarities between single-*emm* clusters.

Steer et al. reported that the African and Asian regions had the greatest diversity of *emm* types (12). This could be due to a variety of factors causing site-tissue tropism and disease manifestation, promoting the dominance of heterologous *emm* types in different regions (31). Our review provides no evidence for marked variation across the continent among most of the more prominent *emm* clusters. When considering the ages of participants infected with GAS, there appears to be no differences compared to that of the overall estimates. There is an increased risk for the transmission of GAS in poorer countries due to household crowding and the lack of income for proper health care (32). Evaluating socioeconomic status among our studies revealed little to no differences in *emm* cluster data.

By clinical manifestation in terms of the invasive nature of the infection, among noninvasive infections, cluster E6 was the most abundant cluster. This is in accordance with previous studies conducted in similarly impoverished areas in India (33, 34) and Brazil (35), which identified *emm* types belonging to cluster E6 (*emm*75 and *emm*81) as the predominant isolates. However, in invasive disease, the predominant *emm* cluster is E3, followed by E6 and D4, which was comparable to the *emm* cluster data shown in Southern Taiwan (16). A study conducted on invasive isolates in the United States suggests that clusters E3 and E6 are the third and fifth highest clusters, respectively (36).

In terms of the current 30-valent vaccine (9), with the assumption that the *emm* type prevalence data from the eight included studies could be generalized for the entire continent, vaccine coverage would be 55.92% of strains isolated in Africa. Frost et al. had shown cross-reactive protection of a single *emm* type with the remaining *emm* types within the same cluster, specifically that of E4 (17). Thus, hypothetically assuming that if a single *emm* type in the 30-valent vaccine would provide cross-protection to the remaining isolates within the cluster, an *emm* cluster-based vaccine would then extend coverage to ∼80% protection against GAS (Fig. 4). Of interest, cluster D4, which comprises 28 heterologous *emm* types and ranked high in this analysis, has only a single representation (*emm*83) included in the vaccine. If cross-protection were to occur within clusters, more *emm* types belonging to cluster D4 ought to gain a particular importance for inclusion into new vaccines, especially since D4 (10.9% of isolates) is the fourth-highest abundant cluster within Africa. It is also important to note that coverage extended to invasive isolates was suboptimal (*n* = 219, 54.1%, inclusive of cross-protection afforded to nonvaccine types).

One of the main strengths of this review is attributed to the search of multiple databases, using an African search filter and a robust approach to the meta-analysis of the data. We systematically and purposefully assessed all the data available with no language exclusions or restrictions to a clinical manifestation of disease, using the most recently published standard quality assessment tools for prevalence studies. We also assessed the risk of bias present in the individual articles, showing that the quality was reasonably high, thus allowing for comparisons across the studies. The main limitations of the review are due to the lack of epidemiological data obtained from low- to middle-income countries in Africa, especially given their relatively high burden of GAS infections. The inclusion of more articles reporting on the prevalence of GAS may further assist in distinguishing differences among the geographical locations, ages, and socioeconomic categories. A further limitation to the results of our systematic review is the significant heterogeneity in the prevalence estimates produced in the meta-analysis; however, this is expected when pooling prevalence studies. We made use of the Freeman-Tukey double arcsine transformation to stabilize the variance of primary studies before pooling, thus limiting the impact of studies with either small or large prevalence on the overall pooled estimates as well as across major subgroups (37).

In conclusion, this systematic review provides the latest evidence for the distribution of *emm* clusters of GAS in Africa. We show that there is negligible variation in *emm* clusters with regard to regions, age, and socioeconomic status across the continent. We further report that the current 30-valent vaccine will provide considerable coverage across the diversity of *emm* cluster types in Africa, thus providing direction for future work to include coverage of clusters D4, E2 to E4, and E6, given that they comprise 83% of the total isolates obtained in Africa.

## MATERIALS AND METHODS

This study employed rigorous methods drawn from the scientific techniques and guidelines offered by the Cochrane Collaboration (38) and by reviews published previously (39, 40). The review protocol has been registered in the PROSPERO International Prospective Register of Systematic Reviews as CRD42017062485.

### Review question.

This review asks the following questions. What is the prevalence of GAS *emm* clusters in Africa in the current available literature? Is there variation in *emm* cluster prevalence based on geography, age, clinical manifestation, or socioeconomic status? We further sought to explore the potential coverage of the current 30-valent vaccine using a cluster-based approach.

### Search strategy.

A comprehensive strategy was developed to search electronic databases to maximize sensitivity (Table S1 in reference 19). The search strategies incorporated both free-term text, which is controlled to suit specific databases individually, and medical subject headings (MeSH) adapted to suit each individual database. A combination of terms relating to “*emm* typing,” “*emm* clusters,” “*emm*/M protein,” and “streptococcal diseases” were used, focusing on the African continent by applying the African search filter previously used by Pienaar and colleagues (41). The following databases were searched as of 29 April 2020; PubMed, Scopus, and Google Scholar for gray literature. The search was not restricted to any publication dates or language; however, abstracts had to be clearly written in English for the study to be considered. Published and unpublished data, such as gray literature including theses and conference proceedings, were also considered for inclusion.

### Inclusion criteria.

All studies that described the prevalence of *emm* clusters or *emm* types within a given population were included in the review. Participants were restricted to the African continent but were not discriminated by clinical manifestation of GAS or site of GAS isolation. All laboratory-confirmed GAS isolates were molecularly characterized by the *emm* typing method as developed by Beall et al. (28) and in alignment with the Centers for Disease Control and Prevention (29) to classify GAS according to sequence analysis of the 5′ hypervariable region of the M protein gene.

Two reviewers independently applied the search strategy to the relevant databases in which the titles and abstracts were evaluated to exclude studies that did not describe the prevalence of GAS. Thereafter, full texts of the included titles and abstracts were retrieved and further evaluated against the inclusion criterion (Table S2 in reference 19). A comparison was made between individual lists: if the reviewers’ lists were not concurrent, discrepancies were discussed and an arbitrator (third reviewer) was contacted to resolve any disagreements.

### Exclusion criteria.

Case reports, narrative reviews, opinion pieces, publications lacking prevalence primary data, and those that referenced methodology that was not according to Beall et al. (28) were excluded from the review. Duplicated studies of the same data sets and participants were removed, and the final most recent publication of the data was considered for inclusion.

### Data extraction and management.

Two reviewers extracted data using a standardized data extraction form, and any contradictions were solved through discussion or by a third reviewer. Search results from the databases listed above and published and unpublished studies were managed with Endnote X9 referencing software. Briefly, data extraction consisted of recording the study demographics (number of study participants, the geographical region, age group of enrolled participants, the clinical manifestation of disease, and socioeconomic status) along with the relevant *emm* type/cluster distributions within the population. Socioeconomic status for the study settings was determined at a country level, according to The World Bank (42).

### Quality assessment.

The risk of bias assessment established by Hoy et al. (43) and modified by Werfalli et al (40) was adapted in questions specific for use in this review (Table S3 in reference 19). Using a quantitative scoring system, studies were characterized as being of a low, moderate, or high risk of bias. A study with a low risk of bias is of high quality, and a low-quality study is associated with a higher risk of bias. Assessing the risk of bias informs the evaluation of heterogeneity in the pooled analyses.

### Analysis.

Data synthesis included three steps: (i) characterizing the study demographics, (ii) documenting *emm* types for *emm* cluster calculations, and (iii) assessing potential vaccine coverage. In each study, the prevalence of *emm* types was recalculated by analyzing figures and tables to confirm the authors’ results and findings and to document the numerators and denominators. In older studies, *emm* typing information needed to be updated using the CDC database (44). Where *emm* cluster information was not reported, the CDC classification system was used to augment missing data (https://www.cdc.gov/groupastrep/lab.html) as well as the original cluster descriptions (13).

To calculate potential coverage, three tiers were assessed: (i) M peptides in the vaccine, (ii) *emm* types that have been shown to be cross-opsonized, and (iii) *emm* types that just happen to be in a cluster that is represented by one or more vaccine *emm* types. Quantitative data analysis was completed using Stata version 14.1 (StataCorp, College Station, TX, USA). We applied the Freeman-Tukey double arcsine transformation option using the metaprop routine to describe the combined prevalence estimates of all included studies with the standard error across the unadjusted estimates (37). *emm* cluster distribution was correlated against different variables (resource setting, clinical manifestation, and age group) in each of the studies. Lastly, we determined the theoretical protective coverage by *emm* cluster cross-opsonization for *emm* types included in the M protein-based vaccine (9).
